# Surface Transport Properties of Pb-Intercalated Graphene

**DOI:** 10.3390/ma14247706

**Published:** 2021-12-13

**Authors:** Markus Gruschwitz, Chitran Ghosal, Ting-Hsuan Shen, Susanne Wolff, Thomas Seyller, Christoph Tegenkamp

**Affiliations:** Institut für Physik, Technische Universität Chemnitz, Reichenhainer Str. 70, 09126 Chemnitz, Germany; markus.gruschwitz@physik.tu-chemnitz.de (M.G.); chitran.ghosal@s2014.tu-chemnitz.de (C.G.); steamliquid@gmail.com (T.-H.S.); susanne.wolff@physik.tu-chemnitz.de (S.W.); thomas.seyller@physik.tu-chemnitz.de (T.S.)

**Keywords:** graphene, Pb intercalation, diffraction, STM, photoemission, surface transport

## Abstract

Intercalation experiments on epitaxial graphene are attracting a lot of attention at present as a tool to further boost the electronic properties of 2D graphene. In this work, we studied the intercalation of Pb using buffer layers on 6H-SiC(0001) by means of electron diffraction, scanning tunneling microscopy, photoelectron spectroscopy and in situ surface transport. Large-area intercalation of a few Pb monolayers succeeded via surface defects. The intercalated Pb forms a characteristic striped phase and leads to formation of almost charge neutral graphene in proximity to a Pb layer. The Pb intercalated layer consists of 2 ML and shows a strong structural corrugation. The epitaxial heterostructure provides an extremely high conductivity of σ=100 mS/□. However, at low temperatures (70 K), we found a metal-insulator transition that we assign to the formation of minigaps in epitaxial graphene, possibly induced by a static distortion of graphene following the corrugation of the interface layer.

## 1. Introduction

The outstanding optical and electronic properties of graphene were intensively studied in the past [1,2]. The next level of complexity comprises the assembly of various 2D materials, e.g., to realize superconducting graphene and design new quantum materials [3,4,5]. Among other approaches, the intercalation of elements is a versatile technique to adjust the properties of 2D materials [6].

In this respect, epitaxial graphene grown by sublimation on SiC is an interesting system. Currently, almost defect-free graphene is grown on semi-insulating SiC(0001) substrates, such that quantum transport experiments are directly performed without the need for any complex transfer processes [7,8,9,10]. Secondly, many elements were intercalated below the graphene sheet, forming partly well-defined interface reconstructions with different functionalities [11,12]. With the exception of a few elements, e.g., H and Sn, the intercalation process happens through open edges of graphene [12,13]. For instance, intercalation of H results in full saturation of the dangling bonds at the interface, thus the intrinsic strong n-type doping is considerably reduced with improved charge carrier mobilities [14,15]. In contrast, the intercalation of, e.g., Ca, K, Gd or Yb causes extreme doping and give rise to electronic correlation effects opening the potential for unconventional superconductivity [16,17,18,19,20]. Intermediate doping scenarios were realized, e.g., by means of Au and Ge, where the intercalation of mono- and bilayer results in realization of metallic and semiconducting phases as well as p- and n-type doped graphene, respectively, [21,22,23].

Intercalation of heavy atoms are expected to introduce spin-orbit coupling (SOC) to the 2D electron gas of graphene. In case of Au intercalation, the SOC was seen for the Au-induced interface states, but not in graphene [22]. For epitaxial graphene grown on Ir(111) the Pb intercalation results in the appearance of electronic resonance effects due to the spatially modulated SOC of the Pb reconstruction with respect to graphene [24]. Nevertheless, surface transport experiments for intercalated Pb with epitaxial graphene have not been performed yet.

In contrast, Pb monolayer and submonolayer structures on Si surfaces were intensively studied in the past. For densely packed Pb monolayer structures, the so-called striped incommensurate phase (SIC) [25] and the $\sqrt{7}\times \sqrt{3}$ reconstruction on Si(111) reveal 2D superconductivity with critical temperatures of 1.8 K and 1.5 K, respectively, [26]. The deposition of a densely packed monolayer system on Si(557) revealed 1D conductivity below 80 K and formation of a spin–orbit density wave phase [27,28,29,30].

Recently, the Pb intercalation of buffer layer (BL) structures on SiC(0001) came into the focus of research [31]. DFT calculations suggest that Pb is acting as an electron donor, depending on the adsorption site and amount [32]. In one of the first intercalation experiments, hexagonally arranged bubble-like structures were reported [33]. Thereby, spectroscopy revealed a p-type doping of graphene, where extrapolation of the π-bands intersect around 100 meV above the Fermi energy. More recently, both striped and hexagonally arranged patterns upon Pb intercalation were reported and explained due to Moiré effect of the graphene layer with monolayer structures of Pb(110) and Pb(111) at the interface, respectively [34].

In this work, we studied the Pb intercalation of buffer layer structures on 6H-SiC(0001). In agreement with previous investigations, we also found striped phases, however, formed with a considerably larger amount of intercalated Pb. Angle resolved photoemission clearly revealed almost charge neutral graphene coming along with an extremely low resistivity.

## 2. Materials and Methods

Buffer layer (BL) samples were prepared by heating nitrogen doped 6H-SiC(0001) substrates (Pam Xiamen Semiconductor). After wet-chemical cleaning the substrates were first flattened by hydrogen etching in a 1000 mbar hydrogen atmosphere at a temperature of 1425 ${}^{\circ }$C for 15 min. Thereafter, long-range ordered BL structures showing the characteristic ($6\sqrt{3}\times 6\sqrt{3}$)-reconstruction were obtained by annealing at 1475 ${}^{\circ }$C. Performing the annealing process in a 10${}^{5}$ Pa Ar-atmosphere yields large terrace lengths of about 3 µm [35].

In order to obtain percolated phases after intercalation, multiple cycles of adsorption and subsequent annealing were mandatory. First, 5 monolayers (MLs) of Pb (Aldrich, 99.999%) were deposited at a rate of 1/3 ML min^−1^ onto the BL sample kept at 200 ${}^{\circ }$C. Thereafter the sample was heated for 5 min at 500 ${}^{\circ }$C. In a second cycle 10 MLs of Pb are deposited onto the sample at room temperature. Finally, the sample was heated again for 5 min at 500 ${}^{\circ }$C. The temperatures were measured by a pyrometer.

The structure of the samples was controlled by scanning electron microscopy (SEM), high resolution low energy electron diffraction (SPALEED) and scanning tunneling microscopy (STM, Omicron VT-STM). Although the intercalated samples turn out to be reasonably stable against oxidation, i.e., all structural and spectral features are fully recovered after mild annealing in vacuum, the samples were, when possible, transferred between the UHV systems (all operating at a base pressure of better than $2\times 10^{-8}$ Pa) using an UHV suitcase. The transport experiments were performed by means of a 4-tip STM/SEM system (Omicron Nanoprobe). Details are described elsewhere [36]. In addition, we performed X-ray and angle resolved photoelectron spectroscopy (XPS, ARPES). For XPS monochromatized Al K_*α*_ radiation (1486.6 eV) was used and the photoelectrons were analyzed with a Specs Phoibos 150-MCD analyzer. ARPES measurements were performed with HeII radiation (40.82 eV) using a Specs UVS 300 He-lamp in conjunction with a Specs TMM304 monochromator and a Specs Phoibos 150 analyzer with a 2D-CCD-detector. The sample investigated by XPS and ARPES had to be exposed to air. Annealing at 320 °C in UHV resulted in the desorption of contaminants, e.g., oxygen.

## 3. Results and Discussion

### 3.1. Structural Properties

**Figure 1:**
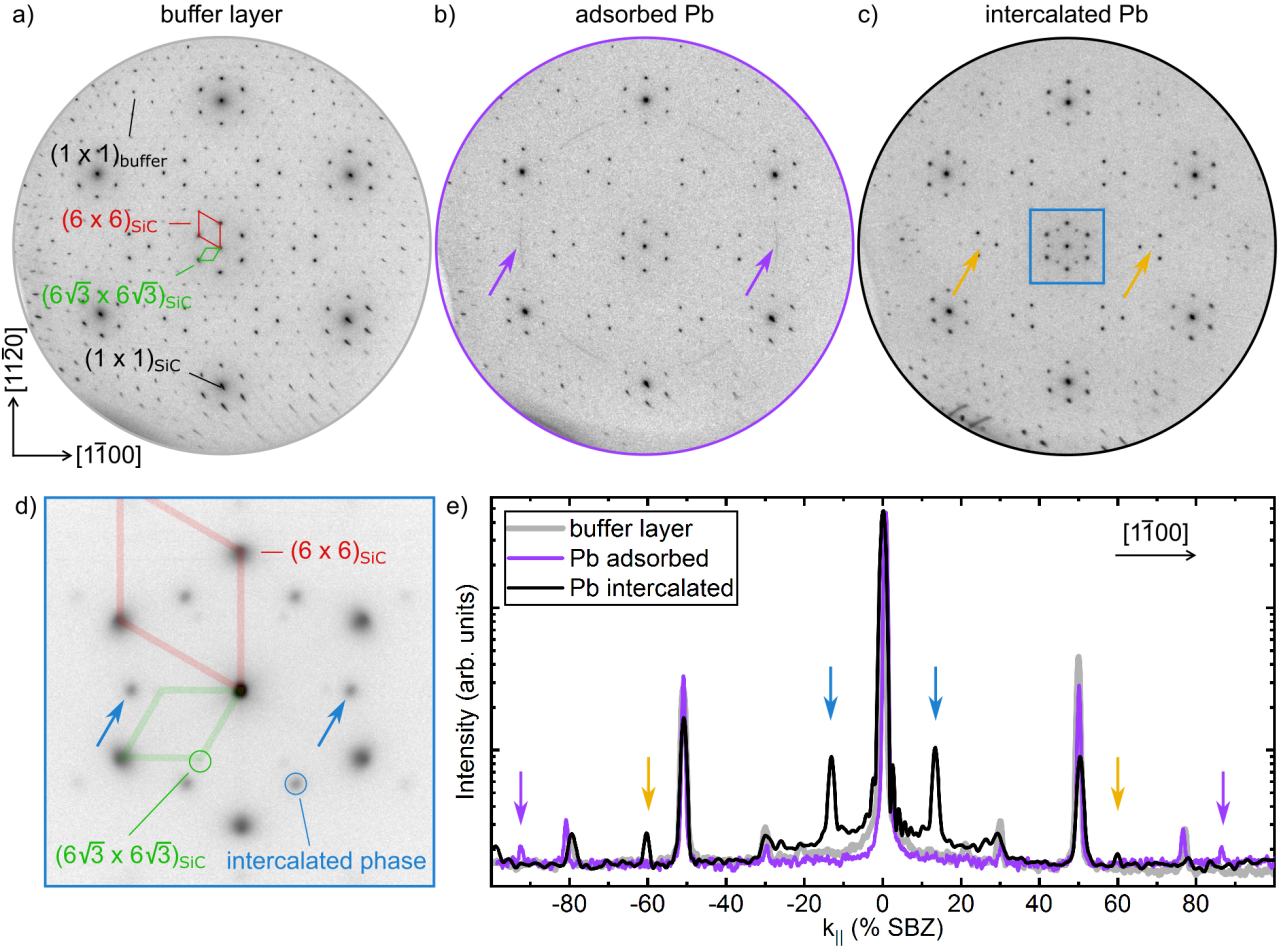
Intercalation process studied by electron diffraction. Sequence of SPALEED images (taken at 100 eV electron energy) of (**a**) pristine BL/SiC(0001), (**b**) after adsorption of 5 MLs of Pb and (**c**) after intercalation of Pb (after adsorption of 5 + 10 ML Pb and annealing cycles). (**d**) Zoom of the (00)-spot after intercalation of Pb (marked in (**c**)). (**e**) Line scans taken along the $\left[1\bar{1}00\right]$-direction for the phases shown in panels (**a**–**c**). The new reconstruction spots upon Pb intercalation are marked by blue and yellow arrows.

Figure 1 displays the process of intercalation investigated by means of SPALEED. The BL/SiC sample in panel a reveals the characteristic $\left(6\sqrt{3}\times 6\sqrt{3}\right)R30^{\circ }$ (6$\sqrt{3}$ in the following) and quasi (6×6) reconstruction spots [37,38]. Deposition of around 5 MLs of Pb on top of the BL results in the formation of rotationally disordered clusters on the surface, as obvious from the new ring-structure (marked by arrows) shown in Figure 1b. The additional spots can be seen better from the line scans taken along the $\left[1\bar{1}00\right]$-direction, shown in panel e. Thereby, the position of the ring in k-space at around 88% of the surface Brillouin zone (SBZ) with respect to the SiC lattice refers to a lateral lattice constant of 3.5 Å, which would resemble one of the lattice parameters of rotationally disordered Pb(110) islands.

After the annealing steps, new diffraction spots appear as shown in Figure 1c and the zoom of the (00)-spot in Figure 1d. The additional diffraction spots are also marked in the line scan shown in Figure 1e. The new intense reflexes are rotated by 30${}^{\circ }$ w.r.t. the (6×6) and are found around the integer spots at 13% SBZ. Thereby, their diffraction intensity depends on electron energy, e.g., at 100 eV and 170 eV they were seen either around the graphene or SiC (not shown), respectively. Simultaneously, the (6×6) and 6$\sqrt{3}$ reconstruction spots of the BL become less intense, but are still present because of an incomplete intercalation. Besides the diffraction peaks induced by the prominent modulation along the $\langle 1\bar{1}00\rangle$ directions, only faint $\left(\sqrt{3}\times \sqrt{3}\right)$-reflexes were seen in LEED (cf. yellow arrow in Figure 1c,e). All other diffraction spots coincide with those expected from the BL phase. The intercalated areas can be increased by multiple adsorption and subsequent annealing steps, extended annealing times and the presence of defects (see below). In the context of the sample shown in Figure 1, an intercalation of up to 70% was achieved via residual defects of the pristine BL/SiC samples.

Before the Pb-induced structure will be analyzed in more detail, we first want to highlight the defect-mediated intercalation process of Pb, such as for the Pb intercalation for graphene on Ru(0001) [39]. Ironically, the continuous improvement for the sublimation process for the growth of BL and graphene on SiC(0001) surfaces [7,40,41,42] makes a generation of further defects, e.g., induced by ion bombardment, mandatory in order to increase the rate of intercalation.

**Figure 2:**
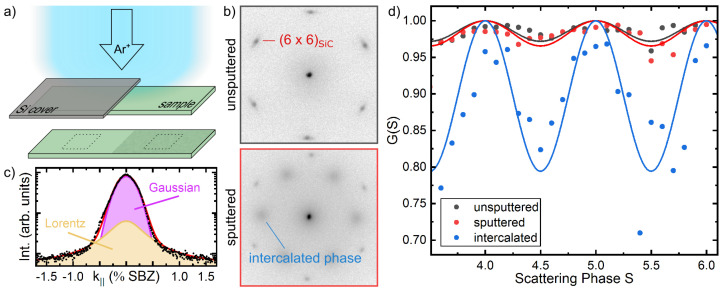
(**a**) Schematic showing the defect generation by Ar-sputtering on one half of the BL/SiC(0001) system. (**b**) SPALEED images of the vicinity of the (00)-diffraction spot after the Pb intercalation procedure (5 ML adsorbed followed by annealing) performed simultaneously on the non-sputtered (top) and sputtered (bottom) part of the sample. (E=100 eV). (**c**) Decomposition of the (00)-spot into a central peak (Gaussian) and shoulder (Lorentzian) (E=114 eV, S=4.4). (**d**) G(S) analysis of the clean and sputtered surface (black, red) and Pb intercalation (blue). The measurements were performed at T=300 K.

The samples were sputtered at ion energies of 1 keV, at a emission current of 10 µA for 10 min at a Ar-pressure of 4 × 10${}^{-4}$ Pa. In order to monitor the quality of the surfaces and possible changes upon sputtering, we performed so-called G(S)-analyses, shown in Figure 2d. Thereby, the ratio of the central diffraction spot with respect to the total intensity, e.g., of the (00)-reflex as depicted in Figure 2c is plotted as a function of the scattering phase $S\approx 2d\sqrt{E(\mathrm{eV})/150.4}$, i.e., different electron energies *E* and in our case for a SiC step height of d=2.5 Å [43]. As mentioned above, the BL/SiC template is characterized by large terraces (see also SEM image below). Therefore, a 2-level system provides a reasonable approach to model the surface roughness within the transfer width of the SPALEED instrument. The G(S) data shown in Figure 2 are well described by $G\left(S\right)=p_{0}^{2}+p_{1}^{2}+2p_{0}p_{1}\cos\left(2\pi S\right)$, where $p_{0}=0.99$ and $p_{1}$ denote the relative surface areas of the two levels ($p_{0}+p_{1}=1$). The value of $p_{0}$ for the initial BL system remains almost unchanged upon sputtering and underlines the gentle penetration of the surface.

By covering the BL/SiC sample with a shadow mask (Si wafer), as sketched in Figure 2a, one half of the sample was exposed to the sputtering. This allows us to study directly the role of defects for the intercalation process. In Figure 2b we show two LEED images around the central spot taken in the sputtered and non-sputtered part of the sample. As obvious, the Pb-induced diffraction spots are visible in the area of increased defect density, while the covered part of the sample remains unchanged. This onset of intercalation is also underlined by the G(S) analysis (cf. blue curve in panel d, revealing $p_{0}=0.93$, i.e., around 7% of the sputtered areas are intercalated. Moreover, the oscillation maxima and minima coincide with the scattering phase calibration done for SiC-steps, i.e., Pb intercalation results also in formation of steps of comparable heights.

**Figure 3:**
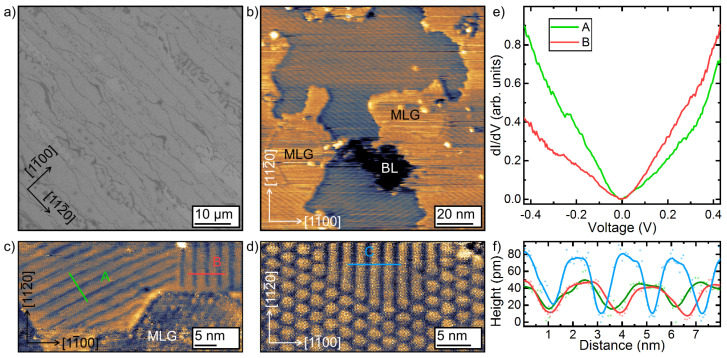
(**a**) Large scale SEM image of the intercalated area. The dark contrast refers to residual (insulating) BL areas. (**b**–**d**) STM images of various phases after intercalation of Pb revealing both striped and bubble-like modulations ((**b**) −0.5 V, 1 nA; (**c**) −0.2 V, 0.02 nA; (**d**) −1 V, 0.2 nA). MLG and BL label non-intercalated monolayer graphene and buffer layer areas, respectively. (**e**) STS spectra on two different striped phases (set point −0.5 V, 0.5 nA). The measurements were performed at LN_2_ temperature. (**f**) Height profiles across various striped phases.

STM measurements were performed on the high-intercalation sample, discussed in context of Figure 1. The majority of phases, which we attribute to the Pb intercalated areas, reveal three kind of stripes rotated by 120${}^{\circ }$ with respect to each other running along the $\langle 1\bar{1}00\rangle$ directions (cf. Figure 3b–d). As is obvious from panel b, around 20% do not show Pb intercalated structures and the Pb intercalated domains are in the order of 50 nm. The stripes are oriented along the zigzag direction of graphene, in agreement with recent STM experiments [34]. Occasionally, a hexagonally arranged bubble-like phase was also seen (Figure 3d). This phase was typically found where at least two striped phases intersect. The latter structure is similar to that reported earlier in ref. [33] for Pb intercalation experiments on MLG samples. From STS measurements shown in Figure 3e, we infer that charge neutral graphene is formed upon Pb intercalation.

The average distance of these stripes is 2.3 nm and fits nicely to the additional diffraction spots at around 13% SBZ, discussed in context of Figure 1. Nevertheless, we also found in our STM experiments smaller and larger distances between the stripes, which seems to be correlated with the amount of intercalated Pb. For instance, the comparably diffuse intensity distribution of the diffraction spot stemming from the modulation in Figure 2b is referred to a stronger variation of these stripe separations.

In ref. [34] the authors showed, that both the striped and hexagonal phase can be well reproduced by assuming Pb(110) and Pb(111) monolayer structures at the interface and explain the STM contrast due to a Moiré effect. However, this is not compatible with the finding of stripes with different distances and widths. Although not all details of the intercalate structure at the interface are known yet, the striped and hexagonal phase reveal some similarities with Pb monolayer reconstructions on Si(111) and Ge(111) [25,44,45,46]. Mainly, the compressive stress within a physical Pb monolayer leads to the formation of various phases, so-called striped and hexagonal incommensurate phases (SIC, HIC) on Si(111) [25]. In this respect, strain effects for Pb monolayers on SiC(0001) with a considerably smaller lattice constant, are likely as well.

### 3.2. Photoemission Spectroscopy

In order to estimate the Pb coverage and check the bonding at the interface XPS measurements were performed. All measurements were made after an additional annealing step. This annealing step was mandatory for cleaning the surface, however, at the expense of a slight desorption of intercalated Pb (around 10%).

**Figure 4:**
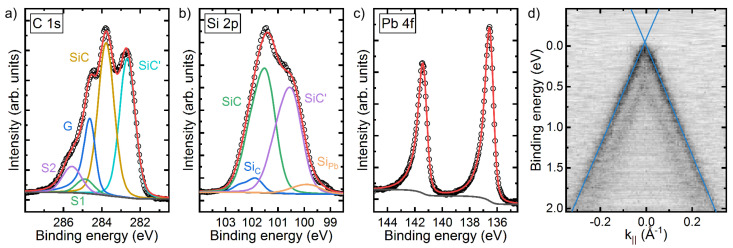
XPS spectra of partially Pb-intercalated BL/SiC. (**a**) C1s, (**b**) Si2p and (**c**) Pb4f state. (**d**) ARPES spectrum around the K-point of graphene. The measurements were performed at T=300 K.

Figure 4a–c shows the core level emissions of the C1s, Si2p and Pb4f states of the intercalated phase, respectively. The spectra are free of oxide-related chemically shifted components. The typical components of a BL, i.e., emissions of SiC (283.8 eV), S1 (284.9 eV) and S2 (285.6 eV), are still visible in the C1s spectra [37]. There, the component SiC belongs to the carbon atoms which are bond in the SiC substrate and stem from the non-intercalated areas. The components S1 and S2 originate from the BL and are due to carbon atoms which are bound only in the BL (S2) or bound additionally to the SiC substrate (S1) [37].

Two further components can be seen in the C1s spectrum. The asymmetrical component labeled G (284.5 eV) is an evidence of successful intercalation. Such an asymmetrical component was also observed in case of the formation of a QFMLG via hydrogen intercalation [14,15,47]. The component SiC’ (282.7 eV) belongs to carbon atoms of the SiC substrate and can be assigned to intercalated areas of the sample. The intensity ratio of the graphene component to the sum of the graphene and BL components refers to an intercalated area of 58%.

A closer look to the Si2p core level in Figure 4b reveals comparable results. The component SiC (101.4 eV of the 3/2 component of the spin–orbit doublet) corresponds to the Si atoms which are only bound in the SiC bulk beneath the BL, whereas the component Si_C_ (101.8 eV) belongs to the Si atoms of the substrate which are bonded to the BL. Two further components can be seen, whereby the component SiC’ (100.4 eV) refers to Si atoms of the SiC substrate beneath intercalated areas of the sample. Finally, the component Si_Pb_ (99.8 eV) can be attributed to the Si atoms which are bond to the intercalated Pb atoms. The energy shift between the SiC and SiC’ components of both, the Si2p and the C1s spectrum, is comparable. Furthermore, the intensity ratio of the SiC’ and SiC component is the same for the C1s and the Si2p spectrum.

Figure 4c shows the Pb4f core level. The spectrum is fitted with an asymmetric doublet and a binding energy of 136.4 eV for the f_7/2_-state which is in good agreement of the bulk value measured for pure Pb [48]. The concomitant metallic behavior is manifested by the strong asymmetry of the peaks due to many-electron interaction effects in metals [49]. From STM measurements we know that all remaining Pb on the sample lies beneath the graphene. Two approaches for estimating the thickness of the intercalated Pb layer have been chosen: (I) A three layer model (from top to bottom: graphene–Pb–SiC) yields to a thickness of $d_{\mathrm{Pb}}=3.3$ Å. This model compares the intensity ratio of the 7/2-peak of the Pb4f core level and SiC’ intensity of the C1s peak with the product of the ratio of the corresponding cross sections, the ratio of the atomic density of Pb and SiC and the weakening of the signal in the Pb layer. This weakening can be described by an exponential function of the ratio of the Pb layer thickness and the inelastic mean free path in Pb. (II) This approach only takes the different signals in the C1s core level into account. A comparison of the intensity ratio of the SiC’ peak and the graphene signal with the intensity ratio of the SiC signal (not intercalated) and the BL components multiplied with the weakening in the Pb layer yields to $d_{\mathrm{Pb}}=6.9$ Å. Again, an exponential function of the ratio of the Pb layer thickness and the inelastic mean free path in Pb describes the reduction in the Pb layer. Furthermore, we take a MLG coverage of 10 % prior to the intercalation into account. Thus, using XPS an average Pb thickness of 5.1 ± 1.8 Å is estimated, which is well above the (111) inter-plane distance of a fcc Pb crystal (2.85 Å), while the first approach was chosen to include two energetically clearly separated peaks, the second one minimizes systematic and energy-dependent errors. Nevertheless, XPS finds prominently multiple layers of intercalated Pb underneath the decoupled graphene supporting the expectations from distinct contrast changes in STM between different areas hosting the characteristic features of intercalation.

In addition, the Pb intercalation was characterized also by ARPES around the K-point of graphene (cf. Figure 4d). Apparently, the Pb intercalation results in the formation of quasi free standing and almost charge neutral graphene. The Dirac point *E_D_* is less than 50 meV above the the Fermi level *E_F_*, yielding a low charge (p-type) carrier density of $p={\left(E_{D}-E_{F}\right)}^{2}/\left(\hbar^{2}v_{F}^{2}\pi \right)=2\times 10^{11}$ cm${}^{-2}$. The spectrum is superimposed by a second Dirac cone. The Dirac point is around 0.5 eV below *E_F_*, thus reminiscent of n-type doped monolayer graphene (MLG) [35]. The BL sample, which we used, was slightly overgrown (10%), i.e., MLG was present at the SiC step edges. Here, this MLG fraction serves as a reference. Moreover, it also seems that Pb intercalation in this area barely took place.

### 3.3. In-Situ Surface Transport Measurements

**Figure 5:**
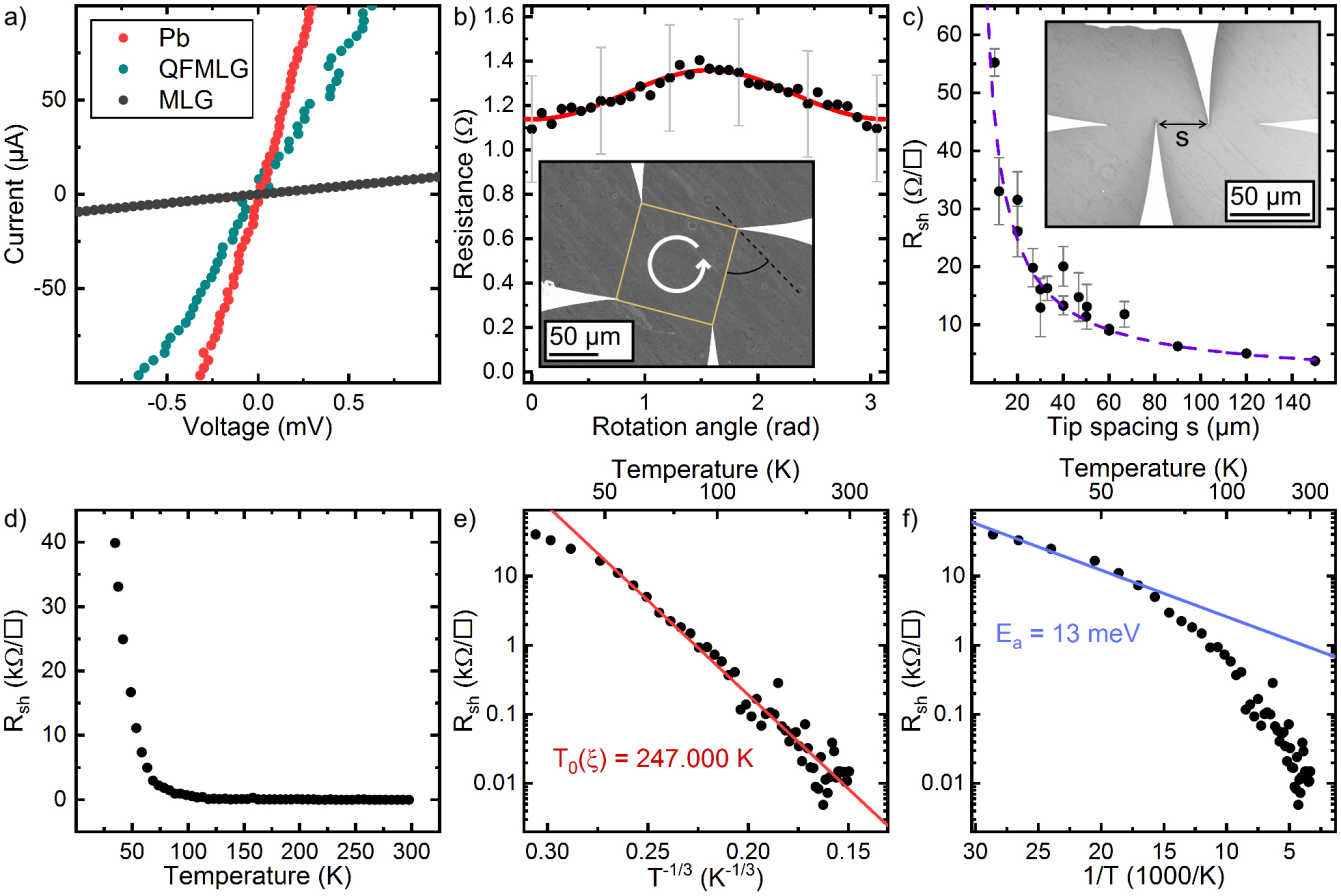
(**a**) IV-curves on Pb-intercalated graphene (T=300 K). For reference, the IV-curves of QFMLG and MLG are shown as well (s=30 µm). (**b**) Rotational square measurement for a fixed tip distance of 100 µm. The inset shows a SEM image of the tip assembly (T=300 K). (**c**) Sheet resistance $R_{\mathrm{sh}}$ as a function of the tip spacing *s*. The inset shows the collinear probe geometry (T=300 K). (**d**) Sheet resistance as a function of temperature for a fixed collinear probe configuration (s=30 µm). (**e**) Anderson plot of the data shown in panel (**d**) covering the temperature range down to 60 K. (**f**) Arrhenius analysis of the transport data shown in panel (**d**). The low temperature regime reveals an activation energy of 13 meV.

Moreover, in situ four-point probe surface transport experiments were performed. As shown in Figure 5a the Pb-intercalated graphene provides linear IV-curves reminiscent of a metallic behavior measured at 300 K. The resistance measured for the Pb-intercalated samples is much lower than those obtained for MLG or QFMLG [50]. In fact, the sheet resistance $R_{\mathrm{sh}}$ for the intercalated graphene is as low as 10 Ω/□, i.e., the charge carrier mobility is within the Drude model $\mu =9\times 10^{5}$ cm${}^{2}$V${}^{-1}$s${}^{-1}$, assuming that only the small hole concentration *p* in graphene, determined by ARPES (see above), is attributing to the transport. For epitaxially grown graphene on Ge/Si(100) similarly high charge carrier mobility values were found [36]. Despite the structural imperfections, mainly the charge neutrality in graphene is responsible for this comparably high value. For comparison, the carrier mobilities in MLG with a concentration of 10${}^{13}$ cm${}^{-2}$ is around 1000 cm${}^{2}$V${}^{-1}$s${}^{-1}$ [15,50]. Nevertheless, we expect in our case that also the Pb interface is contributing.

Rotational square measurements, shown in panel b performed with an average tip distance of 100 µm reveal an extremely low anisotropy ($\rho_{⊥}/\rho_{\left|\right|}=1.08$), i.e., the intercalation process was homogeneous on this length scale. However, the expected 2D-transport signature strongly changes when varying the tip distances. As shown in Figure 5c the sheet resistance decreases reciprocally with the distance of the probes.

In fact, a 1/s-dependency of the resistance with increasing distance is characteristic for 3D transport in a *homogeneous* material [51]. For an isotropic 2D system, the increase of the resistance with increasing distance *s* along the 1D collinear 4 point probe assembly is exactly compensated by lateral spread of the electrons, thus the resistance is not dependent on *s*. In our case, the doped 6H-SiC(0001) substrate is electrically insulating at these temperatures [50,52], thus the 1/s-dependence must be related to the structure of our intercalated 2D phase and were found for nanowire network structures as well [53,54]. Apparently, by increasing the tip distance, the number of percolated electron paths is overcompensating the 1D contribution. This points towards a percolated network of conductive areas. Since we started out with a BL sample, which is electrically insulating, only the intercalated areas result in formation of graphene in agreement with STM and ARPES. As shown above, the intercalation process is strongly triggered by defects, therefore the intercalation process results in formation of Pb islands (cf. Figure 3). The islands are separated either by grain boundaries or patches of the BL. This discrimination is important towards the interpretation of the transport data, since in case of grain boundaries, activated electronic transport between adjacent Pb islands is probable.

Temperature dependent transport measurements for a fixed collinear probe geometry with s=30 µm are shown in Figure 5d. Closer inspection reveals that the R(T)-curve is composed of two areas. There is slight increase in the sheet resistance down to around 70 K. This regime can be nicely described by $\exp{\left(T_{0}/T\right)}^{1/3}$, reminiscent of Anderson localization in a (quasi) 2D system (cf. Figure 5e). The Anderson temperature can be approximated by $T_{0}=14/\left(\xi^{2}k_{B}D\left(E_{F}\right)\right)$ [55,56], where ξ and $D(E_{F})$ denote the correlation length and density of states at the Fermi energy, respectively.

As seen, charge neutral graphene is formed where intercalation takes place. Thereby, Pb grain boundaries are easily overgrown by graphene, resembling an extended band transport channel in parallel to a hopping channel across the Pb islands. In contrast, extended BL areas in between are electrically insulating, thus providing no transport channels. At 300 K we found a highly conductive path for electrons across the surface. Thereby, most likely both channels along the graphene and Pb islands are involved. The Anderson temperature for the fit shown in Figure 5e is $T_{0}=2.5\times 10^{5}$ K, thus for quasi charge neutral graphene correlation lengths of 50 nm results in fair agreement with the average size of the intercalated areas shown in Figure 3. Similar localization effects were reported for weakly hydrogenated graphene [57,58].

The low temperature regime below 70 K can not be captured by disorder along the intercalated path. The Arrhenius analysis in this temperature regime reveals an activation energy of around 10 meV, as shown in Figure 5f. The temperature dependency of only epitaxial graphene can not explain the strong increase of the resistance below 70 K. Measurements on QFMLG and MLG suggest remote-phonon scattering, i.e., the sheet resistance is increasing with increasing temperature [15,50]. In our case, the proximal coupling to the intercalated Pb layer may induce a small gap. Thereby, both the SOC, expected to be present in monolayer structures of Pb, e.g., Pb/Si(111) [46], or the break of the inversion symmetry in graphene due to the structural modulation might be responsible for the gap opening [59]. The STS curves shown in Figure 3e resemble the density of states of intact and charge neutral graphene. However, these measurements were done at LN_2_, i.e., at lower temperatures the proximity coupling may become more severe.

## 4. Summary and Conclusions

In summary, we have comprehensively studied the intercalation of Pb on BL structures on SiC(0001). Triggered by structural defects, up to 70% of the areas was intercalated coming along with the formation of epitaxial graphene on top. In contrast to previous studies, the amount of Pb is around 2 ML, thus the striped phase resemble a structural modulation of the interface rather than a Moiré effect. By means of in situ surface transport the conductivity within these films were determined. Assuming only transport across the graphene, the charge neutrality of the delaminated graphene would come along with charge carrier mobilities of around $9\times 10^{5}$ cm${}^{2}$V${}^{-1}$s${}^{-1}$ at 300 K. Here we expect that also the transport occurs along the Pb monolayer structure. At low temperature, a metal-insulator transition was found, which we relate to gap opening in graphene. However, we failed so far to realize similar Pb structures on SiC and most likely only in presence of epitaxial graphene the striped and bubble-like Pb phase can be stabilized. In order to assign the charge carrier mobilities correctly, knowledge of the atomic details of the Pb structure is mandatory and will trigger our future research.
